# Molecular Characterization of Methicillin-Resistant Staphylococci from the Dairy Value Chain in Two Indian States

**DOI:** 10.3390/pathogens12020344

**Published:** 2023-02-17

**Authors:** Tushar K. Dey, Bibek R. Shome, Samiran Bandyopadhyay, Naresh Kumar Goyal, Åke Lundkvist, Ram P. Deka, Rajeswari Shome, Nimita Venugopal, Delia Grace, Garima Sharma, Habibar Rahman, Johanna F. Lindahl

**Affiliations:** 1 Department of Biosciences, International Livestock Research Institute, Nairobi 00100, Kenya; 2 Zoonosis Science Center, Department of Medical Biochemistry and Microbiology, Uppsala University, 75123 Uppsala, Sweden; 3 ICAR-National Institute of Veterinary Epidemiology and Disease Informatics, Bengaluru 560064, India; 4 Eastern Regional Station, ICAR-Indian Veterinary Research Institute, Kolkata 700037, India; 5 Dairy Microbiology Division, National Dairy Research Institute, Karnal 132001, India; 6 International Livestock Research Institute, Regional Office for South Asia, New Delhi 110012, India; 7 Department of Microbiology, M.S. Ramaiah College of Arts, Science, and Commerce, Bengaluru 560054, India; 8 Food and Markets Department, Natural Resources Institute, Chatham ME4 4TB, UK; 9 Department of Clinical Sciences, Swedish University of Agricultural Sciences, 75007 Uppsala, Sweden

**Keywords:** Methicillin resistance, MRSA, MRCoNS, dairy, milk, food safety, farmers, vendors

## Abstract

Bovine milk and milk products may contain pathogens, antimicrobial resistant bacteria, and antibiotic residues that could harm consumers. We analyzed 282 gram-positive isolates from milk samples from dairy farmers and vendors in Haryana and Assam, India, to assess the prevalence of methicillin-resistant staphylococci using microbiological tests, antibiotic susceptibility testing, and genotyping by PCR. The prevalence of genotypic methicillin resistance in isolates from raw milk samples was 5% [95% confidence interval, CI (3–8)], with 7% [CI (3–10)] in Haryana, in contrast to 2% [CI (0.2–6)] in Assam. The prevalence was the same in isolates from milk samples collected from farmers [5% (*n* = 6), CI (2–11)] and vendors [5% (*n* = 7), CI (2–10)]. Methicillin resistance was also observed in 15% of the isolates from pasteurized milk [(*n* = 3), CI (3–38)]. Two staphylococci harboring a novel *mecC* gene were identified for the first time in Indian dairy products. The only SCC*mec* type identified was Type V. The staphylococci with the *mecA* (*n* = 11) gene in raw milk were commonly resistant to oxacillin [92%, CI (59–100)] and cefoxitin [74%, CI (39–94)], while the isolates with *mecC* (*n* = 2) were resistant to oxacillin (100%) only. All the staphylococci with the *mecA* (*n* = 3) gene in pasteurized milk were resistant to both oxacillin and cefoxitin. Our results provided evidence that methicillin-resistant staphylococci occur in dairy products in India with potential public health implications. The state with more intensive dairy systems (Haryana) had higher levels of methicillin-resistant bacteria in milk.

## 1. Introduction

Antimicrobial resistance (AMR) has become an important public health challenge, especially in low- and middle-income countries (LMIC) [1,2]. Resistant strains may be transmitted via animal-source food from livestock to humans, although evidence of direct links between AMR emergence in humans from food consumption is limited [3]. Antibiotics are widely used as therapeutics, metaphylactics, prophylactics, or as growth enhancing agents in animal production in LMICs [3,4,5], while non-therapeutic usage is less common in developed countries [6,7]. Antibiotics may also be added to preserve perishable foods [8]. Antibiotics used in farm animals often belong to the same classes of antibiotics used in humans [9], thus posing a risk of resistance transmission between animals and humans [10,11]. The use of antibiotics is predicted to rise, especially in LMICs, due to an increased demand for animal products [12]. While new antibiotics are developed, they invariably elicit resistance sooner or later [13,14].

Some pathogenic bacteria found in livestock are zoonotic, and the development of antibiotic resistance in these bacteria is likely to spread to humans through the food chain [15]. Infections caused by resistant bacterial strains in humans are on the rise, including infections caused by *Staphylococcus* spp., *Escherichia coli* [4], *Salmonella* spp. [16], and *Campylobacter* spp. [17]. Staphylococci cause mild to severe sickness in humans [18], more particularly in those whose immune system is weak [19]. They also cause important diseases in dairy animals, such as mastitis, udder impetigo, and wound infections [20,21,22,23]. In veterinary settings, a major concern is the growing spread of methicillin-resistant *Staphylococcus aureus* (MRSA) [24], and methicillin-resistant coagulase-negative staphylococci (MRCoNS) could also constitute a reservoir for genetic determinants of methicillin resistance, giving rise to MRSA [25]. Hence, they pose a threat to human health either through the food supply chain or by directly transmitting resistance genes between humans and animals [24,26].

Methicillin was developed in 1959 as the first semisynthetic penicillin to combat *S. aureus* strains resistant to penicillin [27]. Within a year of its introduction, methicillin-resistant staphylococci were reported [28]. Staphylococcal cassette chromosome *mec* (SCC*mec*) typing is critical for defining clones of methicillin-resistant staphylococci [29]. SCC*mec* are the mobile genetic element genes encoding for PBP2a, which can be transmitted from one bacterial species to another [30].

India is the largest producer of milk globally, and the issues of MRSA and MRCoNS in dairy farms remains a great challenge [31,32]. Both MRSA and MRCoNS have been increasingly detected in dairy animals suffering from mastitis [33,34], and they have the potential to transfer between animals and humans [35,36]. Methicillin resistance along the dairy value chain has been investigated in a few small studies that have exclusively examined milk collected at the farms, and milk at processing centers [37,38,39], but the prevalence of MRSA/MRCoNS has not been investigated in milk from the point of sale or in milk intended for human consumption in India. However, studies from Iran and Saudi Arabia have reported methicillin-resistant bacteria in raw milk, pasteurized milk, and in milk products meant for consumption [40,41,42]. It is noteworthy that the dairy value chain in India is largely informal [43,44] with milk sold by traditional milkmen and vendors who collect the milk from individual farmers and sell it to the consumers [45,46]. The milk sold by these traditional milkmen is often raw and unprocessed, whereas the formal segment consists of cooperatives and private dairies that sell pasteurized packaged milk [47].

In this study, milk samples were collected from two different points of the dairy value chain, one from dairy farmers and the other from dairy vendors in two Indian states: Assam and Haryana. The dairy sector in Assam is mostly non-organized, where 97% of the total milk production passes through unorganized market actors [48]. On the other hand, in the dairy sector in Haryana, intensive farming predominates, and the dairy sector is more organized than in Assam [49]. The objective of this study was to understand the prevalence of methicillin-resistant staphylococci in milk intended for human consumption. The study also aimed to understand differences between the two states, and between farmers and vendors, with a focus on the risk to consumers, and therefore both pasteurized milk and raw milk were included.

## 2. Results

### 2.1. Isolation of Bacteria

The collected milk samples (*n* = 328) were added to a selective medium for isolation of staphylococci, resulting in a total of 329 suspected staphylococci (including duplicates) obtained from 319 milk samples, while the remaining nine milk samples did not result in any isolated bacteria (Table 1 and Table S1). The isolated colonies were initially identified as presumptive staphylococci based on colony morphology, gram-staining, mannitol fermentation, pigment formation, and gelatinase activity using a selective medium. In total, 282 isolates were analyzed further by disc diffusion, molecular screening, and epsilometer testing. The results for raw and pasteurized milks are shown separately.

### 2.2. Antibiotic Susceptibility Testing of Isolates from Raw Milk

In order to identify phenotypic methicillin resistance, we performed an antibiotic disc diffusion test on 282 of the 329 isolates (the remaining 47 isolates were not tested due to shortage of consumables). Twenty of these 282 isolates were from pasteurized milk samples, while the other 262 came from raw milk samples.

We found that 69% [CI (60–78)] of the isolates from raw milk were resistant to oxacillin, with no significant differences between the two states. However, a significantly (*p* ≤ 0.001) higher proportion of isolates from Haryana were resistant to cefoxitin [41%, CI (33–49)] as compared to isolates from Assam [25%, CI (18–35)] (Table 2).

A significantly (*p* < 0.001) higher proportion of isolates from farmers [79%, CI (70–86)] were resistant to oxacillin than from vendors [57%, CI (49–65)]. However, there was no significant difference regarding resistance to cefoxitin (38% and 32%, respectively) (Table 2).

A higher proportion of isolates from Haryana [35%, CI (27–43)] were resistant to both the tested beta-lactam antibiotics (oxacillin and cefoxitin), as compared to the isolates from Assam [23%, CI (15–32)], and more isolates from farmers [36%, CI (27–45)] were resistant to both the antibiotics than isolates from vendors [25%, CI (18–33)] (Table 3).

### 2.3. Molecular Characterization of Isolates from Raw Milk

All the raw milk isolates (*n* = 262) were further subjected to molecular characterization by polymerase chain reaction (PCR) as genotyping method. Overall, 71% [(*n* = 187), CI (65–77)] of the isolates were identified as staphylococci (Table 4). The remaining 29% [(*n* = 75)] of the isolates, which were non-staphylococci, were not further identified as the isolates did not harbor any resistance genes and studying them further was beyond the scope of the study. There were significantly (*p* < 0.001) more staphylococci identified among the isolated bacteria from vendors [(78% (*n* = 113), CI (70–84)] than from farmers [63% (*n* = 74), CI (58–73)].

The prevalence of methicillin resistance defined by genotyping isolates from raw milk isolates was 5% [(*n* = 13), CI (3–8)], with 7% [(*n* = 11), CI (4–13)] in Haryana and 2% (*n* = 2) [CI (0.2–6)] in Assam. The methicillin-resistant determinants *mecA* (*n* = 9) and *mecC* (*n* = 2) were detected in isolates from milk from Haryana, whereas only *mecA* (*n* = 2) was detected in isolates from milk from Assam. Further, *mecA* was more common in staphylococci from vendors [5% (*n* = 7), CI (2–10)] as compared to isolates from farmers [3% (*n* = 4), CI (0.6–6)]. The *mecC* was detected only in isolates from farmers [1% (*n* = 2), CI (0.9–9)] (Table 4).

### 2.4. SCCmec Typing

All the staphylococci with *mecA* gene were screened for SCC*mec* by a multiplex PCR. In Haryana, 33% [(*n* = 3), CI (7–70)] staphylococci with *mecA* were found to be of type V, while in Assam both the staphylococci with *mecA* were of type V. The SCC*mec* type V was found in 57% [(*n* = 4), CI (18–90)] of the staphylococci with *mecA* in milk from vendors, in contrast to 25% [(*n* = 1), CI (0.6–81)] of the staphylococci with *mecA* from milk from farmers (Table 4).

The confirmed staphylococci from Assam showed more often resistance to oxacillin [73% (*n* = 60), CI (62–82)], as compared to staphylococci from Haryana [69% (*n* = 72), CI (59–77)], although not significant. However, significantly (*p* = <0.001) more staphylococci from Haryana were resistant to cefoxitin [39% (*n* = 41), CI (30–49)] than from Assam [15% (*n* = 12), CI (7–24)], respectively (Table 5).

The staphylococci found positive for *mecA* and *mecC* genes by PCR were compared with the result of disc diffusion test to check their antibiotic resistance profile. The majority of the staphylococci with the *mecA* gene were resistant to oxacillin [91% (*n* = 10), CI (59–100)] and cefoxitin [73% (*n* = 8), CI (39–94)] (Table 6). In addition, 36% [(*n* = 4), CI (21–73)] of the staphylococci with the *mecA* gene were found resistant to both oxacillin and cefoxitin. However, both the staphylococci with *mecC* [100% (*n* = 2), CI (15–100)] were found resistant only to oxacillin (Table 6).

We further identified the confirmed methicillin-resistant staphylococci (carrying *mecA* or *mecC* genes) at a species level by a multiplex PCR and found that *Staphylococcus epidermidis* and *S. aureus* were the most common, followed by *S. sciuri,* and *S. arlettae*. Both the isolates from Assam were identified as *S. epidermidis* (Table 7). Both the methicillin-resistant staphylococci carrying *mecC* were identified as *Staphylococcus pseudoxylosus*. The findings of two isolates of *Staphylococcus pseudoxylosus* with the *mecC* gene are novel for India.

Among the confirmed methicillin-resistant staphylococci, most were found to be MRCoNS [73% (*n* = 8), CI (39–94)] followed by MRSA [27% (*n* = 3), CI (6–61)] in isolates from milk from Haryana, while only MRCoNS [100% (*n* = 2), CI (16–100)] were found in the isolates from milk from Assam. The MRCoNS were quite common in isolates from milk from farmers and vendors, whereas MRSA was only found in isolates from milk from vendors.

### 2.5. Epsilometer Test (E-Test)

The genotypically confirmed methicillin-resistant staphylococci were further investigated using the E-test to determine the minimum inhibition concentration (MIC) of the respective drug required to inhibit/kill the bacteria. All the staphylococci with *mecA* gene in milk from farmers (*n* = 6) were found resistant to oxacillin by the E-test as compared to the disc diffusion test, where 5/6 were found resistant to oxacillin. Similarly, all the staphylococci with the *mecA* gene in milk from vendors (*n* = 11) were resistant to cefoxitin by the E-test rather than the disc diffusion test (9/11). In contrast, 12/13 confirmed methicillin-resistant isolates were found resistant to oxacillin and 9/13 isolates were found resistant to cefoxitin by disc diffusion testing (Table 8).

### 2.6. Assessment of Pasteurized Milk Samples from Vendors

There were twenty pasteurized milk samples from vendors from Haryana. All the pasteurized milk samples showed bacterial growth. Among the isolates (*n* = 20) from pasteurized milk, 90% [*n* = 18, CI (68–99)] were identified as staphylococci; however, only 20% [*n* = 4, CI (5–43)] of the isolates were found to harbor methicillin-resistant genes (*mecA*) by PCR genotyping. The confirmed methicillin-resistant staphylococci were identified further at the species level as *Staphylococcus aureus* (*n* = 2) and *S. warneri* (*n* = 1). The fourth isolate with the *mecA* gene was identified as *Enterococcus gallinarum.* The coincidental finding of *Enterococcus gallinarum* with a *mecA* gene is novel for India, but since it was not a staphylococcus, it was not analyzed further. SCC*mec* Type V was found among the two staphylococci with the *mecA* gene, whereas one staphylococcus with the *mecA* gene was untypable. All the confirmed staphylococci with the *mecA* gene in pasteurized milk were found resistant to cefoxitin by E-test and resistant to both oxacillin and cefoxitin by disc diffusion testing.

## 3. Discussion

This study reports the presence of antibiotic resistance in staphylococci from milk from two Indian states, Assam and Haryana. These two states are very different in level of dairy sector development, with Assam being less developed than Haryana. We also compared AMR in milk from different value chain actors, farmers, and vendors, and the presence of AMR in raw and pasteurized milk.

Overall, the level of methicillin resistance in raw milk in our study was lower [5%, (*n* = 13), CI (3–8)] than previously reported for India (13−17%) [50,51], probably because earlier studies were conducted mainly on cows with clinical and subclinical cases of mastitis [40,50,51,52], while the milk collected in our study was from a sale point and intended for consumption. We found that methicillin resistance was higher in Haryana than in Assam [7% (*n* = 11), CI (3–10)] versus [2% (*n* = 2), CI (0.2–6)]. This indicates more intensive dairy production could be associated with higher levels of antibiotic resistance; however, further studies including additional Indian states are needed to confirm this. The proportion of methicillin resistance was the same (5%) in isolates from milk from farmers [5% (*n* = 6) CI (2–11)] and vendors [5% (*n* = 7), CI (2–10)]. The finding of methicillin-resistant staphylococci resistant to both the antibiotics in our study is a cause for concern, as the treatment of choice may lose its effectiveness.

There are no earlier reports of methicillin resistance in milk from vendors in Haryana and Assam; however, a study in another state of India (Andhra Pradesh) reported a prevalence of phenotypic cefoxitin resistance of 5% in milk from vendors [53], much lower than our findings (32% cefoxitin resistance), which raises concerns that resistance to important antibiotics is increasing, and that use of antibiotics must be better regulated in food-producing animals [4,54].

The occurrence of staphylococci in raw milk was found to be 71% [(*n* = 187), CI (65–77)] with 75% [(*n* = 82) CI (65–82)] in Assam and 69% [(*n* = 82) CI (61–76)] in Haryana. However, the vendors’ milk more often contained staphylococci [78% (*n* = 113), CI (78–84)] than the farmers’ milk [64% (*n* = 74), CI (54–72)], which could be attributed to poor hygiene, poor transportation facilities, and improper storage of milk resulting from inadequate sanitation and lack of knowledge among milk handlers regarding the production of safe milk [55,56]. However, the actual source of contamination in milk needs further detailed studies in order to establish the role of value chain actors [57,58].

Most methicillin-resistant staphylococci identified were MRCoNS in both Haryana (*n* = 9) and Assam (*n* = 2). MRSA was comparatively less common and was only present in the isolates from milk of vendors from Haryana (*n* = 5). This finding indicates dominance of coagulase-negative staphylococci as compared to coagulase-positive staphylococci in the dairy milk. Most earlier studies focused on the presence of MRSA as the primary causative agent of mastitis in dairy animals [59,60], but only a few studies have identified MRCoNS as a causative agent of mastitis in dairy cattle [26,61], or as a foodborne health hazard [61,62]. Coagulase-negative staphylococci (CoNS) were formerly thought to be bacteria with very low pathogenicity because they were only described in cases of sub-clinical mastitis, and hence received little attention [63]. However, the mastitis rate in dairy cows by CoNS has been steadily rising during recent years [64], and now it has emerged as a significant animal pathogen [65]. Among the confirmed methicillin-resistant staphylococci, the most dominant species identified was *S. epidermidis,* consistent with earlier reports [66,67]. Our findings suggest that methicillin-resistant staphylococci in milk may constitute an animal disease problem, with resultant treatment expenditure costs and lower milk output [68], as well as being of potential public health importance.

We also found the presence of methicillin resistance in isolates from pasteurized milk [15%, (*n* = 3)] sourced from milk vendors from Haryana. However, the sample size for pasteurized milk in our study was small and the confidence interval large, and hence more milk samples should be studied in order to draw conclusions about the safety of pasteurized milk. The discovery of MRSA and MRCoNS in pasteurized milk suggests post-pasteurization contamination, likely the result of inadequate cooling and hence bacterial growth. Another possibility is that some staphylococci are heat resistant and survive pasteurization [41]. Further studies on the safety of pasteurized milk are needed to identify the extent of the problem where contamination may be introduced post-pasteurization, and ways to minimize the same.

We detected two methicillin-resistant staphylococci, identified as *S. pseudoxylosus*, harboring the *mecC* gene, which is the first report of the *mecC* gene in staphylococci from livestock samples in India.

Among the methicillin-resistant staphylococci with *mecA* (*n* = 11) determinants in raw milk, the most common phenotypic resistance was observed towards oxacillin [92% (*n* = 10), CI (59–100)], followed by cefoxitin [74% (*n* = 8), CI (39–94)]. These results are in line with already reported antibiotic susceptibility testing by disc diffusion for the genotypically confirmed methicillin-resistant staphylococci in milk from three South Indian states [61]. There were four staphylococci with *mecA* gene [36% (*n* = 4), CI (21–73)] that were resistant to both the tested beta-lactam antibiotics (oxacillin and cefoxitin), while the two staphylococci with the *mecC* gene showed resistance towards one antibiotic (oxacillin) only. We also found that all the staphylococci with the *mecA* (*n* = 3) gene in pasteurized milk were resistant to both oxacillin and cefoxitin.

We found that the staphylococci with the *mecC* gene (*n* = 2) were resistant to oxacillin by both disc diffusion test and E-test, which showed efficiency of both the phenotypic tests in detecting the *mecC* gene among the isolated bacteria. Overall, when the results of the disc diffusion test and the E-test were compared with the genotypically confirmed methicillin-resistant staphylococci, our results found the E-test to be more in accordance with the presence of *mecA* or *mecC* genes in staphylococci as compared to the disc diffusion test. Our results are similar to the findings of Wu et al. and Gupta et al. that demonstrated that the E-test is the gold standard method for detecting methicillin resistance [69,70] rather than disc diffusion testing. Our finding also supports the use of both oxacillin and cefoxitin in disc diffusion testing to prevent false negatives and that cefoxitin alone is not reliable in predicting the presence of the *mecA/mecC* gene, also reported by Wu et al. [69].

The SCC*mec* elements are highly diverse and have been classified into 13 different types [71], and there are earlier reports of SCC*mec* type I, III, IV, and V in milk from India [39,61,72]. The only mobile genetic element identified in our study was SCC*mec* type V, which was common among the methicillin-resistant staphylococci in milk from both the states, possibly indicating a common link of resistance gene transfer. The SCC*mec* type V was more among the staphylococci with the *mecA* gene in milk from vendors than in milk from farmers. As SCC*mec* plays a core role in antimicrobial resistance characteristics, molecular epidemiology, and evolution of MRSA [73], a complete overview of the prevalence and structural properties of SCC*mec* is vital for global surveillance and implementation of mitigation efforts against MRSA [74].

Study limitations were the small sample size and the fact that pasteurized milk was only tested in one Indian state, Haryana. Of the total 329 isolates, 47 isolates could not be analyzed due to unavailability of antibiotic discs during the laboratory analyses, and thus removed from further analyses. The non-staphylococci isolates were not identified further. Only the genotypically confirmed methicillin-resistant staphylococci were subjected to the E-test, using oxacillin and cefoxitin for the isolates from farmers’ and vendors’ milk.

Modern and industrial farming systems in LMIC frequently employ high levels of antimicrobials in agriculture and animal husbandry [75] and this practice needs to be regulated. In-depth research is required to better understand the roles played by value chain actors in the establishment of AMR and to determine the root cause and distribution of antibiotic resistance in milk. This will help in understanding the AMR epidemiology in the dairy sector. Correct detection and early diagnosis of methicillin-resistant staphylococci, which has been associated with animal-to-human infection or food poisoning cases, are vital. In addition, the regulation of antibiotics important for animal and public health, with stricter periodical surveillance, would be useful. However, effective surveillance, monitoring of antibiotic consumption, and antibiotic resistance measures present considerable challenges in LMICs due to a lack of capacity, adoption, and integration [76]. Thus, the recent AMR surveillance initiative from the Indian Council of Agricultural Research (ICAR), in the form of the Indian Network for Fisheries and Animal Antibiotic Resistance (INFAAR), is a welcome step [77].

## 4. Materials and Methods

### 4.1. Ethics Statement

Ethical approval for the study was granted by the Institutional Research Ethics Committee (IREC) of the International Livestock Research Institute (ILRI) on 21 September 2015 (No. ILRI-IREC2015-12) and 27 February 2017 (No. ILRI-IREC2017-05) and approved by the collaborating institutes from the Indian Council of Agricultural Research.

### 4.2. Sample Collection

**Figure 1:**
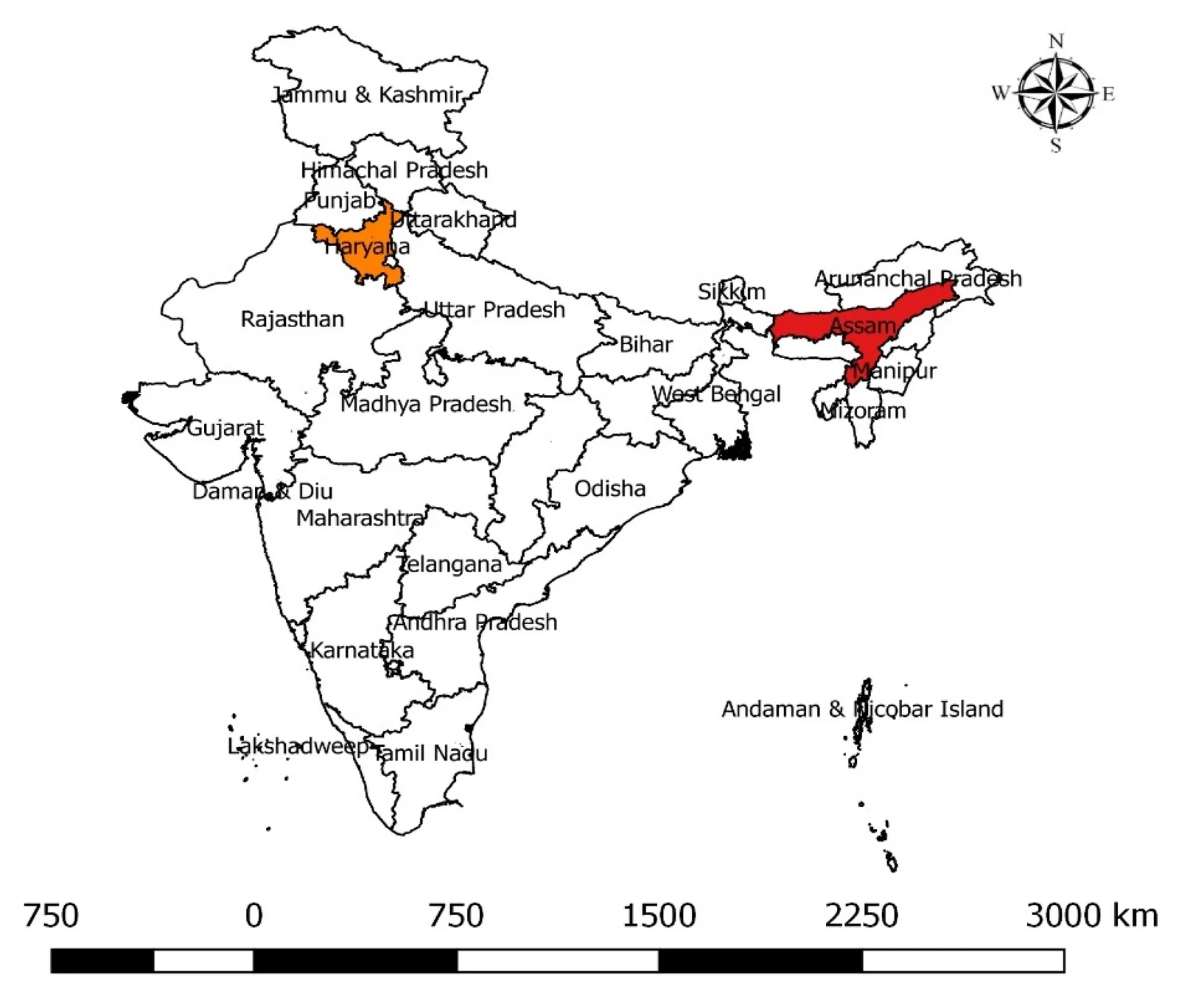
Indian map depicting the sampling states, in highlights, of Assam and Haryana.

A cross-sectional study was conducted in two Indian states, namely Haryana and Assam (Figure 1), during December 2016 and November 2017, and 328 milk samples were collected. Raw milk samples were collected from dairy farmers (*n* = 169) and dairy vendors (*n* = 139) in both the states while pasteurized milk samples were only collected from milk retail outlets/grocery shops (*n* = 20) in Haryana (Table 1). Milk samples from farmers were collected from the districts of Karnal, Bhiwani, and Kaithal in Haryana and the districts of Golaghat, Baska, and Kamrup in Assam during December 2016-February 2017. Milk samples from vendors were collected from the districts of Karnal, Bhiwani, and Kaithal in Haryana (raw milk and pasteurized milk), and the districts of Golaghat, Baska, Kamrup, and Kokrajhar in Assam (raw milk only) during September–November 2017. Even though the number of pasteurized milk samples was low, they represent a risk for consumers, and were therefore included.

**Figure 2:**
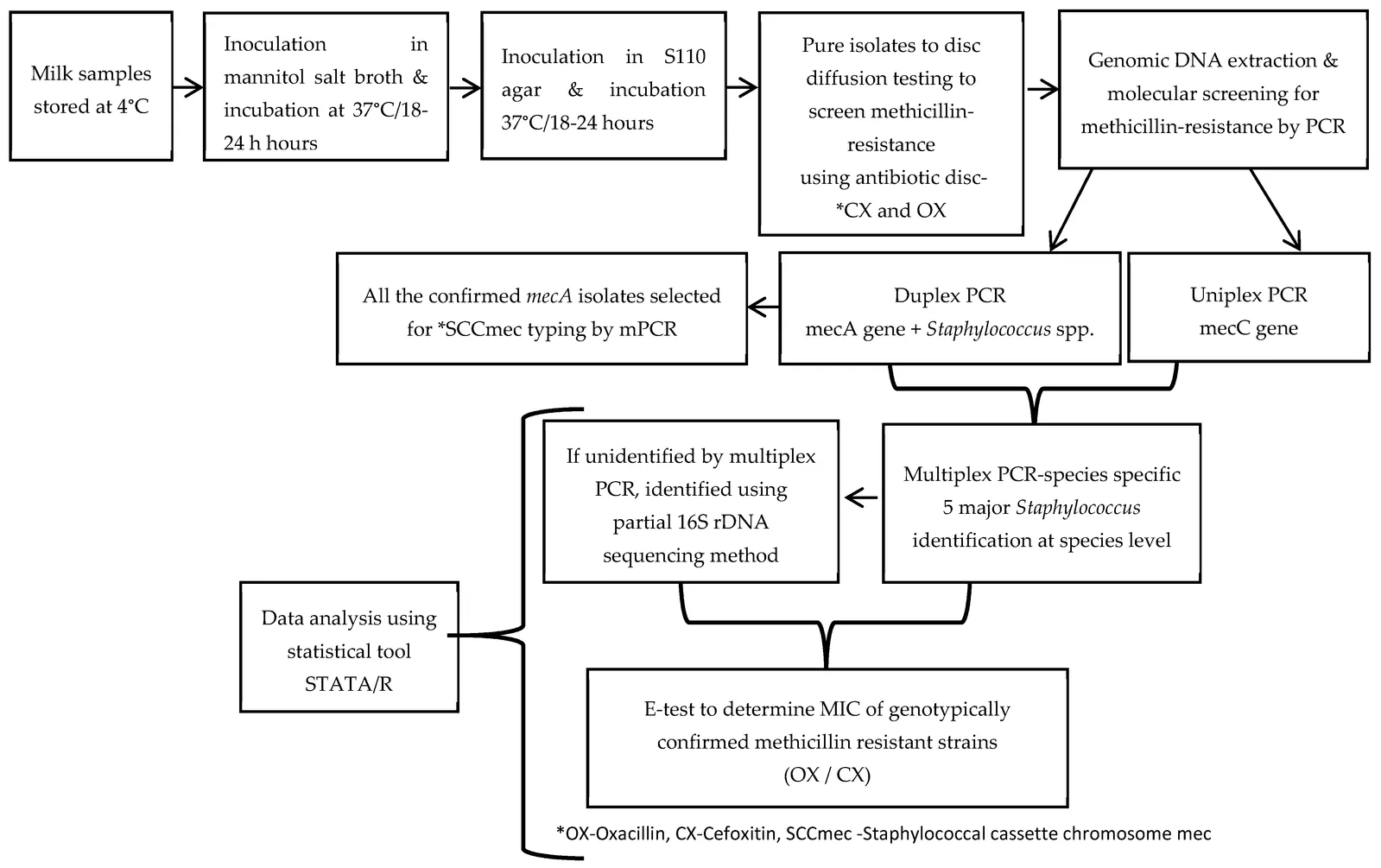
Flow chart for isolation of staphylococci, screening of methicillin-resistant *Staphylococcus aureus*/methicillin-resistant coagulase negative staphylococci using disc diffusion test and molecular method, followed by E-test for the confirmed methicillin-resistant isolates.

A multi-level, random selection of villages and dairy farms was conducted, as well as milk traders and vendors in the same villages, as described in detail elsewhere [78,79]. Milk was sampled from the bulk milk kept at the farm for consumption or sale, or from vendors for sale, in order to represent the milk consumed by consumers to investigate the risk to public health. The farm milk was collected in sterile 50 mL Falcon tubes (Tarson, Kolkata, India). From the vendors, a packaged milk pouch was purchased. The collected samples were transported to the laboratory maintaining a cold chain, and maintained at 4 °C until processing, for isolation of staphylococci using standard laboratory protocols (Figure 2).

The sample size calculation was made assuming 15% of samples had resistant bacteria and by using a 1-sample binomial calculation, assuming 95% level of confidence and 5% precision in the estimates, resulting in about 200 samples per state; to account for a small design effect, we aimed for 240 samples [80]. Given the low numbers of pasteurized samples, the power was very low to detect differences.

### 4.3. Isolation of Bacteria

The samples were initially inoculated in mannitol salt broth and incubated at 37 °C for 18–24 h to isolate presumptive staphylococci. The culture broth was then inoculated in Staphylococcus Agar No. 110 (Hi-media, Maharashtra, India) and incubated at 37 °C for 18–36 h to grow staphylococci. Brain Heart Infusion agar (Hi-media, Maharashtra, India) was used for purification and maintenance of the cultures.

### 4.4. Antibiotic Susceptibility Testing (AST)

Antibiotic susceptibility testing (AST) was performed by the Kirby–Bauer disc diffusion method following the guidelines of the Clinical and Laboratory Standards Institute (CLSI) [81,82]. Prior to AST, a bacterial cell suspension in normal saline solution (0.85%) was made and the turbidity was set to 0.5 McFarland [83]. A sterile cotton swab was dipped into the broth culture tube and rotated several times to get an adequate amount of culture and uniformly spread on the surface of the Mueller–Hinton Agar (MHA) (Hi-media, Maharashtra, India) plates. The antibiotic (Hi-media, Maharashtra, India) cefoxitin (30 µg) and oxacillin (1 µg) discs were placed on the cultured MHA plates. Within 15 min of placing the antibiotic discs on the cultured plates, the plates were incubated at 37 °C for 18–24 h. The plates were then examined for confluent growth and circular zones of inhibition around the antibiotic discs were measured according to the manufacturer’s instruction. For oxacillin and cefoxitin, a zone of inhibition of ≤21 mm and 24 mm for *S. aureus* and CoNS, respectively, were considered as resistant [81]. ATCC 25923-*Staphylococcus aureus* was used as quality control. In the present study, the antibiotic disc diffusion testing was performed for 282 isolates out of the total 329 isolates. The remaining 47 isolates could not be analyzed due to unavailability of antibiotic discs during the laboratory analyses, and thus removed from further analyses.

### 4.5. Molecular Characterization

Genomic DNA was extracted using a DNA extraction kit (Qiagen, Germantown, MD, USA) for all the phenotypically resistant isolates by disc diffusion testing. The concentration and purity of DNA was determined using the nanodrop (Nanodrop 2000/2000c-Thermo Scientific, Waltham, MA, USA). The extracted DNA was subjected to genotyping by duplex PCR, for simultaneous detection of genus staphylococci and methicillin-resistance *mecA* gene, and a uniplex PCR was used for detecting a divergent *mec* gene: *mecC*. The confirmed staphylococci harboring methicillin resistance to either *mecA* or *mecC* genes were further identified at species level by a pentaplex PCR, by which five major staphylococcal species (*S. aureus, S. chromogenes, S. haemolyticus, S. epidermidis*, and *S. sciuri*) can be identified. When samples were found negative by pentaplex PCR, a partial 16S rRNA PCR sequencing method was followed by basic local alignment search tool (BLAST) analysis for identification at species level. Primers used (Table 9) in the study were custom synthesized (Eurofins, Bangalore, India).

### 4.6. Staphylococcus Cassette Chromosome (SCCmec) Typing

The staphylococcus cassette chromosome (SCC*mec*) typing was performed for those staphylococci that were found positive for the *mecA* gene. The SCC*mec* is a combination of two multiplex PCRs; one is a ccr multiplex PCR for typing the ccr complexes, which detects the *mecA* gene and the cassette recombinase complexes, and the other is a *mec* multiplex PCR for typing the *mecA* gene complexes using primers described before [29,73] (Table 10).

### 4.7. Epsilometer Test (E-Test)

All the confirmed methicillin-resistant staphylococci via the PCR genotyping method were subjected to an E-test to determine the minimum inhibition concentration (MIC) required to inhibit/kill the bacteria [81]. To perform an E-test, a bacterial cell suspension was made in normal saline solution (0.85%) and the turbidity was set equivalent to a 0.5 McFarland [83]. A sterile cotton swab was dipped into the broth culture tube and rotated several times to get adequate amount of culture; it was then uniformly applied on the surface of the MHA (Hi-media, Maharashtra, India) plate. The antibiotic cefoxitin (0.016–256 mcg/mL) and oxacillin (0.016–256 mcg/mL) (Hi-media, Maharashtra, India) strips were placed on the MHA agar plate, using a sterile forceps, by gently pressing the antibiotic strips to ensure their complete contact with the surface of the agar plate. The inoculation was performed within 10–15 min of the inoculum being prepared in normal saline. The plates were then incubated at 37 ℃ for 16–20 h, and then examined for the MIC value from the scale in terms of µg/mL where the ellipse edge intersects the strip. For *S. aureus*, oxacillin ≥ 4 and cefoxitin ≥ 8 were considered as resistant, and for CoNS, oxacillin ≥ 0.5 was considered as resistant [88]. For quality control, *S. aureus* ATCC 29213 was used.

### 4.8. Statistical Analysis

Statistical tests were performed using STATA 15.1 (STATACorp, Texas College Station, TX, USA). The chi square test and Fischer exact test were used to test association between variables. A *p*-value below 0.05 was considered as statistically significant. The isolates were considered phenotypically methicillin-resistant if the isolates were resistant to both or either of the two tested beta-lactam antibiotics, oxacillin and cefoxitin, by using the disc diffusion test. However, the isolates were confirmed as methicillin-resistant if the isolates harbored either the *mecA* or *mecC* genes by PCR genotyping. In our study, only 282 isolates of the 329 isolates from the milk samples were tested by an antibiotic disc diffusion test. The missing data for 47 isolates [(*n* = 43) Haryana and (*n* = 4) Assam)] were removed from further statistical analysis.

## 5. Conclusions

This study found methicillin-resistant staphylococci in milk intended for human consumption, which has public health implications. The more frequent occurrence of antibiotic-resistant genes in Haryana suggests that levels of resistance are higher in more intensive and industrialized dairy systems. This underscores the need for stricter antibiotic usage control on commercial intensive farms. In addition, better understanding of the vendors’ role in procuring and quality assurance of milk is needed. We recommend adherence to pasteurization techniques, improving vendor and farmer practices, and sensitizing all dairy value chain actors on the importance of AMR. That even pasteurized milk is contaminated with staphylococci harboring methicillin-resistant genes is of great concern; however, our study included only a few pasteurized samples. We demonstrated the occurrence of staphylococci harboring the *mecC* gene in milk for the first time in India. The only SCCmec type identified in milk from Haryana and Assam was Type V, presumably indicating a common link of resistance gene transmission. The phenotypic test in our study supports that cefoxitin alone is unreliable for predicting the *mecA/mecC* genes and suggest using both oxacillin and cefoxitin in disc diffusion testing to prevent false negatives.

## Figures and Tables

**Table 1:** Details of milk samples; number of samples positive in bacterial culture and bacterial isolates.

Milk Source	Sample Type	Assam	Haryana	Total
Milk from dairy farmer	Raw milk	43	126	169
	Samples positive	43	117	160
	Isolates	47	117	164
Milk from dairy vendor	Raw milk	63	76	139
	Samples positive	63	76	139
	Isolates	67	78	145
	Pasteurized milk	0	20	20
	Samples positive	0	20	20
	Isolates	0	20	20
	Total samples	106	222	328
	Total positive	106	213	319
	Total isolates	114	215	329

**Table 2:** Isolates of presumptive staphylococci from raw milk showing the antibiotic resistance profile by a disc diffusion test.

**Antibiotics**	**Isolates in Milk from Haryana (*n* = 152)**			**Isolates in Milk from Assam (*n* = 110)**			***p*-Value**
	Resistant % (CI ^#^)	Intermediate % (CI ^#^)	Sensitive % (CI ^#^)	Resistant % (CI ^#^)	Intermediate % (CI ^#^)	Sensitive % (CI ^#^)	
Oxacillin	99 65.13 (57–73)	0	53 34.87 (27–43)	76 69.09 (60–78)	0	34 30.91 (22–40)	0.510
Cefoxitin	62 40.79 (33–49)	5 3.29 (1–8)	85 55.92 (48–64)	28 25.45 (18–35)	0	82 74.55 (65–82)	0.002
**Antibiotics**	**Isolates in Milk from Farmer (*n* = 117)**			**Isolates in Milk from Vendor (*n* = 145)**			***p*-Value**
	Resistant % (CI ^#^)	Intermediate % (CI ^#^)	Sensitive % (CI ^#^)	Resistant % (CI ^#^)	Intermediate % (CI ^#^)	Sensitive % (CI ^#^)	
Oxacillin	92 78.63 (70–86)	0	25 21.37 (14–30)	83 57.24 (49–65)	0	62 42.76 (35–51)	<0.001
Cefoxitin	44 37.61 (29–47)	4 3.42 (0.9–9)	69 58.97 (50–68)	46 31.72 (24–40)	1 0.69 (0.01–4)	98 67.59 (59–75)	0.163

^#^ 95% confidence interval (CI).

**Table 3:** Isolates of presumptive staphylococci from raw milk showing resistance to either or both the antibiotics by a disc diffusion test.

Phenotypic Methicillin-Resistance	Isolates in Milk from Haryana State (*n* = 152)	Isolates in Milk from Assam State (*n* = 110)
	Resistant % (CI ^#^)	Resistant % (CI ^#^)
Resistant to at least one antibiotic	106 69.74 (62–80)	79 71.82 (62–80)
Resistant to both oxacillin and cefoxitin	53 34.87 (27–43)	25 22.73 (15–32)
Phenotypic methicillin-resistance	Isolates in milk from farmer (*n* = 117)	Isolates in milk from vendor (*n* = 145)
	Resistant % (CI ^#^)	Resistant % (CI ^#^)
Resistant to at least one antibiotic	94 80.34 (72–87)	91 62.76 (54–71)
Resistant to both oxacillin and cefoxitin	42 35.90 (27–45)	36 24.83 (18–33)

^#^ 95% confidence interval (CI).

**Table 4:** Identification of genus staphylococci, methicillin-resistant (*mecA/mecC*) genes, and SCCmec typing among the isolated bacteria from raw milk by genotyping.

Milk Source	Staphylococci % (CI ^#^)	*p*-Value	*mecA* Gene % (CI ^#^)	*p*-Value	*mecC* Gene % (CI ^#^)	*p*-Value	SCC*mec* Type V ^&^ % (CI ^#^)	*p*-Value
Milk from Haryana (*n* = 152)	105 69.08 (61–76)	0.406	9 5.92 (3–10)	0.210	2 1.32 (0.1–7)	0.837	3/9 33.33 (7–70)	0.545
Milk from Assam (*n* = 110)	82 74.55 (65–82)		2 1.82 (0.2–6)		0		2/2, 100 (15–100)	
Milk from farmer (*n* = 117)	74 63.25 (54–72)	0.013	4 3.42 (0.9–8)	<0.001	2 1.71 (0.2–6)	<0.001	1/4, 25.00 (0.6–81)	0.697
Milk from vendor (*n* = 145)	113 77.93 (70–84)		7 4.83 (2–10)		0		4/7, 57.14 (18–90)	

^#^ 95% confidence interval (CI); SCCmec—staphylococcal cassette chromosome; ^&^ All the *mecA* positive staphylococci were subjected to SCCmec typing.

**Table 5:** Antibiotic resistance among the confirmed staphylococci isolated from raw milk.

Methicillin-Resistance by Disc Diffusion Test	Staphylococci in Milk from Haryana State (*n* = 105)	Staphylococci in Milk from Assam State (*n* = 82)	*p*-Value
	Resistant % (CI ^#^)	Resistant % (CI ^#^)	
Oxacillin	72 68.57 (59–77)	60 73.17 (62–82)	0.521
Cefoxitin	41 39.05 (30–49)	12 14.63 (7–24)	<0.001

^#^ 95% confidence interval (CI).

**Table 6:** Antibiotic resistance profile among the confirmed methicillin-resistant staphylococci (with *mecA/mecC* genes).

Methicillin Resistance by Disc Diffusion Test	Staphylococci with *mecA* Gene (*n* = 11)	Staphylococci with *mecC* Gene (*n* = 2)
	% (CI ^#^)	% (CI ^#^)
Oxacillin	10 90.91 (59–100)	2 100 (15–100)
Cefoxitin	8 72.73 (39–94)	0
Resistance to both oxacillin and cefoxitin	4 36.36 (21–73)	0

^#^ 95% confidence interval (CI).

**Table 7:** Species-level identification for the confirmed methicillin-resistant staphylococci.

Genotypically Confirmed Methicillin-Resistant Bacteria at Species Level	Staphylococci in Milk from Haryana (*n* = 11)	Staphylococci in Milk from Assam (*n* = 2)	Staphylococci in Milk from Farmer (*n* = 6)	Staphylococci in Milk from Vendor (*n* = 7)
	% (CI ^#^)	% (CI ^#^)	% (CI ^#^)	% (CI ^#^)
*Staphylococcus aureus* *(mecA)*	3 27.27 (6–61)	0	0	3 42.86 (9–81)
*Staphylococcus epidermidis* *(mecA)*	4 36.36 (10–69)	2 100 (15–100)	3 50 (11–88)	3 42.86 (9–81)
*Staphylococcus sciuri* *(mecA)*	1 9.09 (0.2–41)	0	1 16.67 (0.4–64)	0
*Staphylococcus arlettae* *(mecA)*	1 9.09 (0.2–41)	0	0	1 14.29 (0.3–57)
*Staphylococcus pseudoxylosus* *(mecC)*	2 18.18 (2–51)	0	2 33.33 (4–77)	0

^#^ 95% confidence interval (CI).

**Table 8:** Results of E-test and the disc diffusion test for the confirmed methicillin-resistant staphylococci.

Milk Type, Source	Methicillin Resistance	Disc Diffusion Test		E-Test (MIC Value) ^#^	
	*mecA/mecC* Genes (*n* = 13)	Oxacillin	Cefoxitin	Oxacillin	Cefoxitin
Raw milk (Farmer)	*mecA*	R	R	R (3)	-
Raw milk (Farmer)	*mecA*	R	R	R (6)	-
Raw milk (Farmer)	*mecA*	NR	R	R (1)	-
Raw milk (Farmer)	*mecA*	R	NR	R (6)	-
Raw milk (Farmer)	*mecC*	R	NR	R (1)	-
Raw milk (Farmer)	*mecC*	R	NR	R (0.75)	-
Raw milk (Vendor)	*mecA*	R	R	-	R (6)
Raw milk (Vendor)	*mecA*	R	R	-	R (16)
Raw milk (Vendor)	*mecA*	R	R	-	R (6)
Raw milk (Vendor)	*mecA*	R	NR	-	R (8)
Raw milk (Vendor)	*mecA*	R	R	-	R (24)
Raw milk (Vendor)	*mecA*	R	R	-	R (12)
Raw milk (Vendor)	*mecA*	R	R	-	R (50)

R-Resistant, NR-Not Resistant, - Not tested, ^#^ Minimum inhibitory concentration (MIC) in mcg/mL.

**Table 9:** PCR primer details for identifying MRSA/MRCoNS.

Identification	Gene	Sequence (5’-3’)	Annealing Temp (°C)	Amplicon Size (bp)	Remarks	Refs.
*Staphylococcus* spp. Methicillin resistance	*16S rRNA* *mecA*	GTGATCGGCCACACTGGA CAACTTAATGATGGCAACTAAGC ACGAGTAGATGCTCAATATAA CTTAGTTCTTTAGCGATTGC	60	842	Duplex PCR	[84,85]
Methicillin resistance	*mecC*	GCTCCTAATGCTAATGCA TAAGCAATAATGACTACC	56	304	Uniplex PCR	[86]
*S. aureus*	*23S rRNA*	AGCGAGTCTGAATAGGGCGTTT CCCATCACAGCTCAGCCTTAAC	56	894	Multiplex PCR	[87]
*S. chromogenes*	*Soda*	GCGTACCAGAAGATAAACAAACTC CATTATTTACAACGAGCCATGC	58	222		
*S. haemolyticus*	*Soda*	CAAATTAAATTCTGCAGTTGAGG GGCCTCTTATAGAGACCACATGTTA	58	531		
*S. epidermidis*	*Rdr*	AAGAGCGTGGAGAAAAGTATCAAG TCGATACCATCAAAAAGTTGG	56	130		
*S. sciuri*	*Gap*	GATTCCGCGTAAACGGTAGAG CATCATTTAATACTTTAGCCATTG	56	306		

**Table 10:** PCR primer details for staphylococcal cassette chromosome mec typing.

PCR	Gene	Primer Designation	Sequence (5’-3’)	Annealing Temp (°C)	Amplicon Size (bp)	Remarks, Primer Pair	Ref.
mec complex typing	*mecA*	mA7	ATATACCAAACCCGACAACTACA	60			[29]
	*mecI*	mI6	CATAACTTCCCATTCTGCAGATG		1963	mA7-mI6 (class A*mec*)	
	*IS1272*	IS7	ATGCTTAATGATAGCATCCGAATG		2827	mA7-IS7 (class B*mec*)	
	*IS431*	IS2(iS-2)	TGAGGTTATTCAGATATTTCGATGT		804	mA7-IS2(iS-2) (class C*mec*)	
ccr complex typing	*mecA*	mA1 mA2	TGCTATCCACCCTCAAACAGG AACGTTGTAACCACCCCAAGA	57	286	mA1-mA2	
	*ccrA1*	α1	AACCTATATCATCAATCAGTACGT		695	α1-βc	
	*ccrA2*	α2	TAAAGGCATCAATGCACAAACACT		937	α2-βc	
	*ccrA3*	α3	AGCTCAAAAGCAAGCAATAGAAT		1791	α3-βc	
	*ccrB1, ccrB2, ccrB3*	Βc	ATTGCCTTGATAATAGCCTTCT				
	*ccrA4*	α4.2	GTATCAATGCACCAGAACTT		1287	α4.2-β4.2	
	*ccr B4*	β4.2	TTGCGACTCTCTTGGCGTTT				
	*ccrC*	γF	CGTCTATTACAAGATGTTAAGGATAAT		518	γF-γR	
		γR	CCTTTATAGACTGGATTATTCAAAATAT				

## Data Availability

Data is made available from the authors upon reasonable request.

## Supplementary Materials

The following supporting information can be downloaded at https://www.mdpi.com/article/10.3390/pathogens12020344/s1. Table S1: Details of all the milk samples, results of antibiotic-resistance profile for the isolated gram-positive bacteria and their molecular characterization.
